# Second trimester post-abortion family planning uptake and associated factors in 14 public health facilities in Central Uganda: a cross-sectional study

**DOI:** 10.1186/s40834-022-00199-4

**Published:** 2023-01-14

**Authors:** Susan Atuhairwe, Claudia Hanson, Nazarius Mbona Tumwesigye, Kristina Gemzell-Danielsson, Josaphat Byamugisha

**Affiliations:** 1 Department of Obstetrics and Gynecology, Makerere University, Kampala, Uganda; 2 Department of Reproductive Medicine and Infertility, Mulago Specialised Women and Neonatal Hospital, Kampala, Uganda; 3 Department of Global Public Health, Karolinska Institutet, Solna, Sweden; 4 Department of Disease Control, London School of Hygiene and Tropical Medicine, London, UK; 5 Department of Epidemiology & Biostatistics, School of Public Health, Makerere University, Kampala, Uganda; 6 Department of Women’s and Children’s Health, Karolinska Institutet, and the WHO collaborating centre, Karolinska University Hospital, 17176 Stockholm, Sweden

**Keywords:** Family planning, Post-abortion care, Second-trimester, Abortion, Health facilities, Uganda

## Abstract

**Background:**

Post-abortion family planning counselling and provision are known high impact practices preventing unintended pregnancies. Little is known, however, about specific needs in the second trimester. Our study aims to assess post-abortion family planning uptake and its associated factors among women with second-trimester incomplete abortion.

**Methods:**

We conducted a cross-sectional survey of 1191 women with incomplete second trimester abortion that received treatment at 14 comprehensive emergency obstetric care public health facilities in central Uganda from August 2018 to November 2021. We computed the post-abortion uptake of family planning within 2 weeks of treatment, described the types of methods accepted, and the reasons for declining family planning. We described the socio-demographic, reproductive, abortion-related, and health facility characteristics. We used mixed effects generalized linear models to obtain percentage differences for factors independently associated with post-abortion family planning uptake.

**Results:**

Second-trimester post-abortion family planning uptake was 65.6%. Implants (37.5%) and progestin only injectables (36.5%) were the commonly chosen methods; natural (0.1%), permanent (0.8%), and condoms (4%) were the least chosen methods. 45.2% of the women who declined family planning desired another pregnancy soon. Women whose spouses were aware of the pregnancy or had planned pregnancy had 11% (− 10.5, 95% CI − 17.1 to − 3.8) and 12% (− 11.7, 95% CI − 19.0 to − 4.4) less uptake compared to women whose spouses were not aware of the pregnancy or those with unplanned pregnancies respectively. Uptake was 8% (− 7.8, 95% CI − 12.6% to − 3.0%) lower among Islamic women compared to Anglicans. Women who received post-abortion family planning counselling or had more than four live births had 59% (59.4, 95% CI 42.1 to 76.7) and 13% (13.4, 95% CI 4.0 to 22.8%) higher uptake compared to women who did not receive counselling or women with no live births, respectively.

**Conclusions:**

The uptake of second-trimester post-abortion family planning in Uganda was higher than previous estimates. Post-abortion family planning counselling, grand multiparity, and the need to avoid an unplanned pregnancy enhance post-abortion family planning uptake in the second trimester. Ministry of Health should strengthen post-abortion family planning counselling, especially couple counselling; at all health facilities in the country and also ensure an adequate and accessible supply of a wide contraceptive method mix.

**Supplementary Information:**

The online version contains supplementary material available at 10.1186/s40834-022-00199-4.

## Introduction

Globally, an estimated 121 million women experience unintended pregnancy annually, and 61% of these unintended pregnancies end up as abortions [1]. Sub-Saharan Africa has the highest burden of unintended pregnancies (91 per 1000 women), unsafe abortions (77%), and a low contraceptive rate (29%) [2]. Following an abortion, the World Health Organization (WHO) recommends that a woman should wait for at least 6 months before getting pregnant again, to minimize pregnancy-related health risks [3]. Accessible and consistent use of family planning has potential to reduce maternal deaths by an estimated 25–35% [4]; with Indonesia giving estimates as high as 43% [5].

Post-abortion family planning (PAFP) is an essential element of post-abortion care (PAC) [6], that averts repeated unintended pregnancies and promotes healthier timing and spacing of pregnancies. Women are often unaware that fertility may resume within 2 weeks after an abortion hence the importance to ensure access to counseling and a range of modern methods in PAC delivery settings at the initial visit to prevent a subsequent unplanned pregnancy [2, 7]. While this is the ideal, some women may not benefit from either service due to: limited physical space at the facility and time for counselling, lack of family planning methods, misinformation or lack of knowledge on when to start the method, and lack of skills especially in provision of long term and permanent methods [8]. This increases the unmet need for family planning among PAC clients and is seen as a missed opportunity. Women are more likely to accept a family planning method post-abortion if offered appropriate counselling and method provision at the same setting [9].

Studies in Africa and Asia demonstrate PAFP uptake rates ranging from 78% up to 90% [9–11]. There’s limited data on PAFP uptake in Uganda, a country with a low contraceptive prevalence rate of 39% among married women [12], however some evidence shows that not all women who want a family planning method can access the service [12, 13]. A qualitative study in Uganda exploring the role of midwives in PAC highlighted barriers to PAFP uptake as: lack of time for counselling due to heavy work load, inability to access PAFP counselling on the gynaecology ward and misinformation on side effects of family planning [13]. Additional barriers to PAFP include: religious and cultural beliefs [11], and the restricted prescription of long acting reversible contraceptives to doctors [14].

All modern methods can be used after an abortion [3]. The most commonly used methods are the short acting reversible contraceptive methods like pills, condoms, and injectables while the long acting reversible contraceptives, implants and intrauterine devices, have low uptake rates despite their known high efficacy in preventing pregnancies [10, 14]. A recent multi-country survey on abortion-related morbidity in East and Southern Africa showed hormonal injectables and implants as the most frequently used family planning methods post abortion in Uganda [15]. The use of long acting reversible contraceptives is a high impact recommended practice in PAC [7]; due to their high effectiveness and user satisfaction and thus highly recommended compared to self-administered methods like the oral pills and condoms [16], to prevent unwanted pregnancies.

Thus, while possible factors that increase PAFP uptake are well described including history of induced abortion, being parous, age above 24 years, treatment at health centres compared to hospitals, urban facilities, and non-Catholic health facilities [11, 14, 17, 18] the relative importance for second trimester PAFP is understudied. Yet women often have complications that may increase the importance of effective methods. We therefore carried out this study to assess post-abortion family planning uptake and its associated factors in women with second trimester incomplete abortion.

## Methods

### Study design, settings and participants

Our study adhered to the STROBE guidelines for reporting observational studies in epidemiology [19]. We collected cross-sectional data on women’s FP uptake within 2 weeks of second trimester PAC management with misoprostol as part of a multicenter randomized controlled equivalence trial [20]. The trial assessed safety, effectiveness and acceptability outcomes of treatment for second trimester (13–18 weeks) incomplete abortion using misoprostol when provided by midwives versus physicians in Uganda. We conducted the study from August 2018 to November 2021 at 14 public health facilities; comprising of four health centre IVs, eight general and two referral hospitals; in Central region of Uganda. Health centre IVs are the first level health care facilities in Uganda that offer comprehensive emergency obstetric care services. Details of the study setting are described elsewhere [21]. All participants in the primary trial were eligible and included in this study.

### Data collection

Midwives, who received a standardized 2-day PAC training, obtained informed consent and counselled all trial participants on PAFP in a place with auditory and visual privacy before discharge from the health facility. Each counselling session lasted approximately 30 minutes, and covered three aspects [22]:

1. Education and information on: fertility after an abortion, ideal birth interval for women’s reproductive health, advantages and disadvantages of all types of FP methods available and their use including emergency contraception.
2. Provider-guided information on: future plan to use FP methods, experience with previously used method and expectations, beliefs and myths related to FP.
3. Free provision of a FP method of their choice based on WHO medical eligibility criteria 2015 guidelines and verification of their understanding of its use.

As part of PAC, participants returned after 2 weeks for a follow-up review. At the follow-up visit, participants received additional PAFP counselling and could start a method if they had not accepted at the initial visit (when they received emergency treatment) or not started using the method selected at the initial visit. We used a paper-based interviewer administered questionnaire in English language to capture information on women’s sociodemographic, reproductive history, abortion-related, and health facility characteristics. We also obtained information on whether a participant initiated a FP method, the type of FP, and the reasons for decline among participants that did not take up any PAFP method. The questionnaire was developed by the research team after an extensive literature review. We obtained institutional ethical approval and all study participants gave written informed consent.

### Outcomes and explanatory variables

The main outcome, PAFP uptake, was defined as initiating a FP method within 2 weeks of PAC management as self-reported and verified by records in the PAC register. We also collected information on: type of method accepted, and reasons for declining PAFP.

Explanatory variables included self-reported sociodemographic characteristics like age in completed years, marital status, religion, highest level of education attained, occupation, spouse aware of the pregnancy, whether pregnancy was planned, and history of domestic violence in the preceding 12 months. Reproductive characteristics like number of pregnancies and live births, previous history of abortion, whether the current abortion was spontaneous or induced, previous use of contraception, and PAFP counselling were self-reported. Gestational age in weeks (based on clinical examination of uterine size), type of uterine evacuation, and health facility level were abstracted from the clinical records.

### Bias

Given that some variables were very personal and sensitive, we reduced interviewer, response and recall biases by having trained and experienced midwives perform the PAFP counselling and data collection in real time. We also ensured privacy and confidentiality to build participants’ confidence and trust.

### Quantitative variables

The outcome variable PAFP uptake and independent variables like marital status, religion, education level, occupation, spouse aware of the pregnancy, and history of domestic violence were all inherently categorical. Other categorical variables included: current abortion status (spontaneous/induced), whether the pregnancy was planned, previous use of contraception, PAFP counselling, type of uterine evacuation, and health facility level. Age was collected as a continuous variable and number of pregnancies and live births as discrete variables. Based on previous epidemiological distributions, age was categorized– 15-19, 20–24, 25–29, 30–34, and 35–49 years; number of pregnancies as 1, 2–4, and 5–15; and parity as 1, 2–4, and 5–13.

### Statistical methods

Data was entered in Epi-data version 3.1, validated and cleaned then exported to Stata version 14 for analysis. We had minimal missing data (< 1%) and therefore used complete-case analysis. Sociodemographic, reproductive, abortion-related and health facility characteristics; categorical outcomes; and reasons for declining PAFP uptake; were presented with descriptive statistics. Continuous variables were summarized using means (standard deviation) if normally distributed or medians (interquartile range) if not normally distributed, and proportions (95% confidence intervals) for categorical variables. We used generalized linear mixed-effects model (gaussian family and identity link) with co-variates as fixed effects and healthcare facility as a random effect. To ascertain variables independently associated with PAFP uptake, all variables (marital status, religion, spouse aware of pregnancy, planned pregnancy, parity, FP counselling, and type of evacuation) with *p* < 0.2 at bivariate analysis were entered into multivariable generalized linear models. Additional variables (age, health facility level, gestation age, occupation, previous use of FP, and whether the abortion was induced) with biological plausibility were included in the model. We used the backward selection model building technique and obtained adjusted percentage differences of PAFP uptake. Elimination criteria from the model was *p*-value > 0.05. We performed regression diagnostics using the likelihood-ratio test and selected the most parsimonious model. We computed 95% CIs and statistical significance established at *p* < 0.05.

## Results

Of 1191 participants recruited from August 2018 to November 2021 from the 14 study sites, we excluded one participant who had an on-going pregnancy. Participants’ age varied from 15 to 47 years and the mean age was 26.2 (SD 6.0) years. Over one-third (33.4%, *n* = 397) were aged 20 to 24 years. Women were predominantly married or cohabiting (77.1%, *n* = 917), catholic (41.5%, *n* = 494), unemployed (47.5%, *n* = 565), and had attained either secondary (43.5%, *n* = 518) or primary (43.1%, *n* = 513) school as their highest level of education. The spouse was aware of the pregnancy in most cases (89.7%, *n* = 1067), over two-thirds (67.9%, *n* = 808) had planned the pregnancy, and 8.9% (*n* = 106) reported history of abuse within the previous 12 months (Table 1).

**Table 1:** Sociodemographic characteristics of study participants at 14 public health facilities in central region of Uganda, 2018–2021

Characteristic	Number of participants ***N*** = 1190	%
Age (years)	26.2 ± 6.0	
15–19	147	12.3
20–24	397	33.4
25–29	313	26.3
30–34	198	16.6
35–49	133	11.2
Missing data	2	0.2
Marital status		
Married or cohabiting	917	77.1
Single/divorced/widow	273	22.9
Religion		
Anglican	371	31.2
Catholic	494	41.5
Islam	230	19.3
Other (SDA, Born again)	95	8.0
Education		
None	36	3.1
Primary	513	43.1
Secondary	518	43.5
Tertiary	123	10.3
Occupation		
Unemployed	565	47.5
Formal employment	142	11.9
Self employed	483	40.6
Spouse aware of the pregnancy		
Yes	1067	89.7
No	123	10.3
Pregnancy planned		
Yes	808	67.9
No	382	32.1
History of abuse within 12 months		
Yes	106	8.9
No	1084	91.1

Abbreviation: *SDA* Seventh-day Adventist

The median gestation age was 14 (IQR 13 to 18) weeks. Fifty six percent of the women had two to four pregnancies (55.6%, *n* = 661) and 40% of all women had two to four live births (40%, *n* = 476). Two hundred and twenty-eight (19.2%) women reported a previous history of abortion and 105 (8.8%) women had induced the current abortion. Over half (59.5%, *n* = 708) of the participants had previously used FP and nearly all (98.6%, *n* = 1173) received PAFP counselling. Most women received PAC at general hospitals (52.2.%, *n* = 621) and used misoprostol alone (90.9%, *n* = 1082) for uterine evacuation (Table 2).

**Table 2:** Reproductive, abortion-related and health facility characteristics of study participants at 14 public health facilities in central region of Uganda, 2018–2021

Characteristics	Number of participants ***N*** = 1190	%
Gestational age (weeks)^a^	15.0 ± 1.6	
Number of pregnancies	3 (2–4)	
1	281	23.6
2–4	661	55.6
5–15	248	20.8
Number of live births	1 (0–3)	
0	344	28.9
1	260	21.9
2–4	476	40
5–13	110	9.2
Previous history of abortion		
Yes	228	19.2
No	962	80.8
Type of current abortion		
Spontaneous	1085	91.2
Induced	105	8.8
Previous use of FP		
Yes	708	59.5
No	482	40.5
Currently counselled on FP		
Yes	1173	98.6
No	17	1.4
Type of uterine evacuation method		
Misoprostol alone	1082	90.9
Misoprostol & MVA	64	5.4
Misoprostol & D&E	39	3.3
Missing data	5	0.4
Health facility level		
Referral hospital	226	19
General hospital	621	52.2
Health centre IV	343	28.8

^a^Missing data for seven participants

Data are mean ± standard deviation or median (interquartile range)

Abbreviations: *FP* family planning, *MVA* Manual Vacuum Aspiration, *D&E* Dilation and Evacuation

Overall, 781 (65.6%) women received a FP method within 2 weeks of the second-trimester PAC. At least 60.6% of the 1190 women accepted a FP method on the initial visit and 469 (39.4%) declined. Among the 1140 women that returned for the follow-up visit, 819 (71.8%) received additional PAFP counselling. Of the 446 women who had initially declined FP, 60 (13.5%) women accepted at the follow-up visit (Table 3).

**Table 3:** Family planning uptake among second-trimester post-abortion women

Characteristic	Number	%
Accepted FP on first visit		
n	1190	
Yes	721	60.6
FP accepted on follow-up visit among those who had declined FP at first visit		
n	446	
Yes	60	13.5
Received extra counselling on follow-up visit		
n	1140	
Yes	819	71.8
Overall FP uptake within 2 weeks		
n	1190	
Yes	781	65.6

Abbreviations: *FP* Family planning

**Fig. 1:**
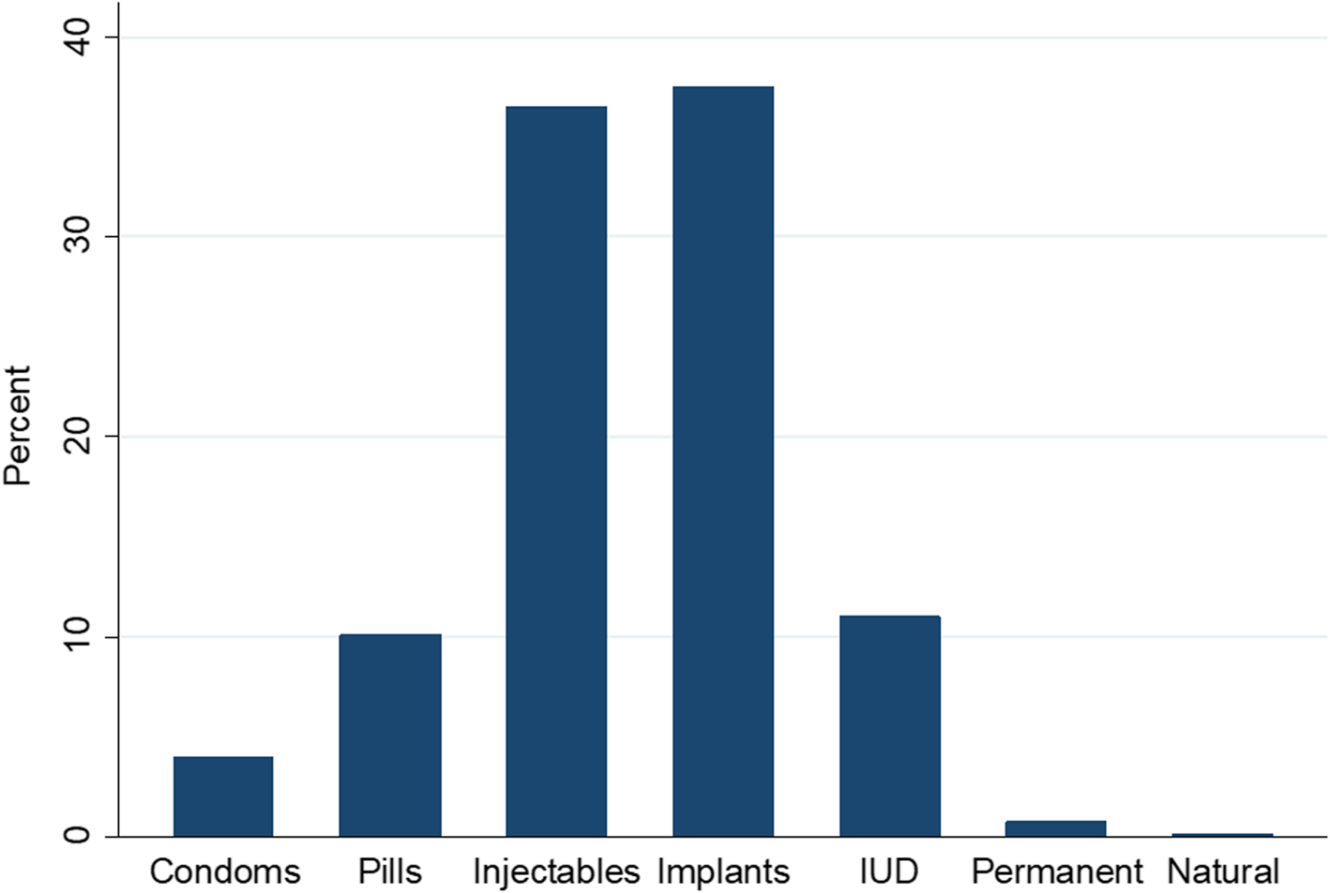
Distribution of second trimester posta bortion family planning uptake, 2018 to 2021

Women commonly chose implants (37.5%, *n* = 293) and progestin only injectables (36.5%, *n* = 285). The least chosen methods were natural [withdrawal] (0.1%, *n* = 1), permanent (0.8%, *n* = 6), and condoms (4%, *n* = 31) (Fig. 1). Almost half (45.2%, *n* = 157) of the women who declined FP wanted another pregnancy soon, 16% (*n* = 57) reported they would choose later, 14.7% (*n* = 51) decided to first consult with a relative, and 12.1% (*n* = 42) were either not interested or students (see Additional file 1).

In our study, religion, spousal awareness of pregnancy, planned pregnancy, PAFP counselling, and parity were the factors independently associated with PAFP uptake (Table 4).

**Table 4:** Bivariate and adjusted model for factors associated with second-trimester postabortion family planning uptake

Characteristic	All women ***N*** = 1190	Accepted FP n (row%)	Unadjusted model*		Adjusted model*^#^	
			Percentage difference (95% CI)	***P*** value	Percentage difference (95%)	***P*** value
Religion						
Anglican	371	255 (68.7)	Reference		Ref	
Catholic	494	331 (67.0)	−1.7 (−6.5 to 3.0)	0.476	−2.2 (− 6.0 to 1.6)	0.265
Islam	230	140 (60.9)	−7.9 (−12.6 to −3.1)	0.001	− 7.8 (− 12.6 to − 3.0)	0.001
Other (SDA, Born again)	95	55 (57.9)	−10.8 (− 23.1 to 1.4)	0.082	− 10.4 (− 21.3 to 0.4)	0.060
Spouse aware of the pregnancy						
Yes	1067	680 (63.7)	−18.4 (−26.3 to − 10.5)	< 0.001	−10.5 (−17.1 to −3.8)	0.002
No	123	101 (82.1)	Reference		Reference	
Pregnancy planned						
Yes	808	494 (61.1)	−14.0 (−22.4 to −5.6)	0.001	−11.7 (−19.0 to −4.4)	0.002
No	382	287 (75.1)	Reference		Reference	
Currently counselled on FP						
Yes	1173	780 (66.5)	60.0 (44.3 to 76.9)	< 0.001	59.4 (42.1 to 76.7)	< 0.001
No	17	1 (5.9)	Reference		Reference	
Parity (live births)						
0	344	213 (61.9)	Reference		Reference	
1	260	162 (62.3)	0.4 (−6.5 to 7.3)	0.912	3.5 (−2.9 to 9.9)	0.278
2–4	476	324 (68.1)	6.1 (−2.7 to 15.0)	0.172	8.9 (0.0 to 17.7)	0.049
5–13	110	82 (74.6)	12.6 (2.9 to 22.3)	0.011	13.4 (4.0 to 22.8)	0.005

*Statistics computed using generalized linear mixed-effects model

^#^Adjusted for religion, spousal awareness of pregnancy, planned pregnancy, currently counselled on FP, and parity

Abbreviations: *FP* Family planning, *CI* Confidence interval, *SDA* Seventh-Day Adventist

Women who were Catholic, Islam, Born Again, or Seventh Day Adventist all had a lower PAFP uptake compared to Anglicans. However significantly, the uptake of PAFP was 8% (− 7.8, 95% CI − 12.6% to − 3.0%) lower among Islamic women compared to Anglicans controlling for spousal awareness of pregnancy, planned pregnancy, parity, and FP counselling. Adjusting for religion, planned pregnancy, parity, and FP counselling, the uptake of PAFP was 11% (− 10.5, 95% CI − 17.1 to − 3.8) less among women whose spouses were aware of the pregnancy compared to women whose spouses were not aware of the pregnancy. Women with planned pregnancies had 12% (− 11.7, 95% CI − 19.0 to − 4.4) lesser uptake of PAFP compared to women with unplanned pregnancies adjusting for religion, spousal awareness of pregnancy, parity, and FP counselling. Keeping religion, spousal awareness of pregnancy, parity, and planned pregnancy constant, the uptake of PAFP was 59% (59.4, 95% CI 42.1 to 76.7) more among women who received PAFP counselling compared to women who did not receive PAFP counselling. Women who had unplanned pregnancies tended not to inform their spouses about the pregnancy (results not shown). Finally, we observed a gradual rise in PAFP uptake with increasing parity. Women with five or more live births had 13% (13.4, 95% CI 4.0 to 22.8%) higher uptake of PAFP compared to women with no live birth, controlling for spousal awareness of pregnancy, planned pregnancy, and FP counselling.

We did not find age, marital status, health facility level, type of uterine evacuation, occupation, previous use of FP, education, history of abuse, previous abortion history, and induced abortion as significant factors for PAFP uptake in our study.

## Discussion

This is the first cross-sectional study from Uganda to assess PAFP uptake and associated factors among women who received second trimester PAC. Our study showed that 65.6% of the women received PAFP within 2 weeks of second-trimester PAC. Implants and progestin only injectable contraceptives were the commonly accepted methods. Majority of the women who declined PAFP desired another conception soon. Factors independently associated with PAFP uptake were religion, spousal awareness of pregnancy, planned pregnancy, parity, and PAFP counselling.

The PAFP uptake of 65.6% in our study is important against the background that typically second trimester PAFP estimates of around 50% are reported from previous studies assessing first and second-trimester abortion. And that women after a second-trimester abortion are typically less likely to accept PAFP compared to first-trimester [23–25]. The higher uptake in our study could be due to: the exclusive focus on second-trimester PAFP, additional PAFP counselling at the follow-up visit, and a free constant supply of FP commodities to ensure a wide contraceptive mix. Almost all previous studies enrolled women in both first and second trimester and majority consistently reported PAFP lower in second trimester compared to first trimester [12, 23, 24]. It is speculated that women with second trimester PAC often have spontaneous abortions and thus desire to get pregnant quickly as a way of coping with the loss. In our study, almost half of the women who declined PAFP wanted to get pregnant quickly. Although previous evidence suggests that women in second trimester may desire to conceive quickly following induced abortion for medical reasons [24]; the type of abortion was not significant in our study. On the other hand, planned pregnancy was significantly associated with increased PAFP uptake in our study, so probably these women may desire to quickly replace the lost pregnancy. Another common reason for refusal was the need to consult the spouse or relative, showing the lack of autonomy women have in their own family planning decision making [7].

In our study, women commonly chose implants (37.5%) and progestin only injectables (36.5%), similar to a recent multi-country survey [15]. Injectables are the most commonly used PAFP method in most African countries with South Africa (88%), Ethiopia (55%), and Malawi (70%) reporting some of the highest rates [25]. In Uganda, injectables are the most commonly accepted contraceptive method among all women (18.5%) [12]. Implants have slowly been gaining ground standing at 6.3% among all married women [12]. The Ministry of Health in Uganda made a deliberate move to encourage the use of LARCs due to their cost effectiveness over time and no required repeated visits to the health care provider. LARCs however, require a trained health care provider for insertion and removal which may act as a barrier, especially in settings with limited staff, supplies, and equipment [23]. Among the LARCs, implants were more frequently chosen probably due to availability of a health care provider for the insertion, free commodity, counselling, their relative ease of method insertion compared to the Intra-uterine device [25]. Health care providers may prefer to insert the Intra-uterine device at a follow-up visit to avoid the higher rate of expulsion observed in both surgical and medical post-abortion insertion [26]. Women may also have a negative attitude towards IUDs due to myths, misconceptions, and fear of side effects [27]. Permanent methods were the least chosen modern PAFP in this study. Similar to a Brazilian study, it is not surprising given that 46% of the women in our study were less than 24 years, and thus unlikely to use a permanent method of FP [14]. With the exception of India and other countries in Asia [25], use of permanent methods is consistently low especially in low-income countries [23]. While the condoms offer dual protection, their uptake in our study was low. Condom use is noted to be high in some areas opposed to hormonal family planning in low-income settings [11]. The liberal acceptance of hormonal methods in our setting [12], could explain the low uptake of the barrier method.

Islamic women were less likely to accept PAFP compared to Anglican women. While Anglicans tend to be more liberal regarding family planning, other religions have more conservative views regarding sexuality and family planning [11]. In countries where women have strong religious affiliations, interpretations of the doctrine have strong implications on their reproductive choices. In our study a woman was less likely to take up PAFP if the spouse was aware of the pregnancy or if the pregnancy was planned. A Brazilian study did not find any association between pregnancy planning and PAFP [14]. Our findings of 11% reduction in PAFP uptake if the spouse was aware of the pregnancy contrast with a systematic review on contraceptive use in Sub-Saharan African which showed that communication with the male partner increased contraceptive uptake [28]. This could indicate a planned pregnancy within a functioning partnership and so more effort in counselling may be put on managing spacing. Grand multiparous women in our study had a greater uptake of PAFP compared to women with no live birth, and our findings agree with studies in similar settings [29, 30]. It is probable that grand multiparous women have experienced pregnancy related morbidities and therefore choose to delay or limit other pregnancies. In addition, frequent interaction with the health care system may expose one to information on family planning with its benefits and hence improve PAFP uptake.

As expected, uptake of PAFP was higher among women who received PAFP counselling in line with results from a recent meta-analysis from Eastern-Africa [22, 24, 29]. In our study, PAFP counselling and method provision were mainly done by midwives who tend to spend more time with admitted patients, therefore building trust and confidence [7]. At most facilities, women initiated the PAFP method at the same service point as PAC which is known to increase acceptance of FP [7], and received additional FP counselling at the follow-up visit. This depicts the importance of comprehensive PAFP counselling together with immediate method provision on uptake of PAFP [22].

A departure of our findings from previous studies is that factors such as age, marital status, health facility level, type of uterine evacuation, occupation, previous use of FP, education, history of abuse, previous abortion history, and induced abortion were not significantly associated with second trimester PAFP uptake [29, 31, 32]. One possible explanation could be that previous studies recruited participants who received first and second trimester PAC, and yet we only recruited women in second trimester.

Our study had both strengths and limitations. We used a large dataset of 1191 second-trimester PAC women. To our knowledge, no study has exclusively documented PAFP uptake in this population of women. Data was captured in real time reducing chances of recall bias and missing data. A limitation is the study was conducted at high patient-load public health facilities and patient characteristics may differ from those in private settings or health facilities with few patients. A second limitation may be social desirability bias given the sensitivity of some questions. To address this issue, all participating midwives received a standardized 2-day training prior to study onset.

This multi-centre study was performed at public health facilities ranging from Health Centre IVs, general hospitals, and referral hospitals in a low-income country making these results generalizable to similar settings.

## Conclusions

Uptake of second-trimester PAFP in Uganda was 65.6% which is higher than reports from previous studies and against the backdrop of PAFP counselling and immediate method provision. Women’s preferred PAFP methods were implants and progestin only injectable contraceptives. PAFP counselling, religion, spousal awareness of pregnancy, planned pregnancy, and grand multiparity were the factors significantly associated with second trimester PAFP uptake. We recommend that Ministry of Health strengthens PAFP counselling at all health facilities in the country that offer PAC services with emphasis on couple counselling, and also ensure an adequate and accessible supply of a wide contraceptive method mix. Using a qualitative approach, we recommend further research exploring facilitators and barriers to second trimester PAFP.

## Supplementary Information

**Additional file 1.**

## Data Availability

The datasets used and/or analysed during the current study are available from the corresponding author on reasonable request.

## Abbreviations

CI: Confidence interval
D&E: Dilation and Evacuation
FP: family planning
MVA: Manual Vacuum Aspiration
PAC: Post-abortion care
PAFP: Post-abortion family planning
SDA: Seventh-day Adventist
WHO: World Health Organization
