# Supplementary material for: Second trimester post-abortion family planning uptake and associated factors in 14 public health facilities in Central Uganda: a cross-sectional study
Source: Contracept Reprod Med. 2023 Jan 14;8:4. doi: 10.1186/s40834-022-00199-4 (PMC9840296; doi:10.1186/s40834-022-00199-4)
Supplement: Supplementary file 1 — Additional file 1. [file 40834_2022_199_MOESM1_ESM.docx]

| **Reason** | **Number**  **N=347** | **%** |
| --- | --- | --- |
| Choose later/partner was not around | 57 | 16.4 |
| Wants pregnancy | 157 | 45.2 |
| Ask husband/mother | 51 | 14.7 |
| Not in relationship | 8 | 2.3 |
| Fear of side effects | 15 | 4.3 |
| Partner refused | 15 | 4.3 |
| Religion prohibits FP | 2 | 0.6 |
| Lacks interest/tired of FP/school | 42 | 12.1 |

**Additional file 1. Reasons for declining family planning among second-trimester postabortion care clients**
